# Challenges and Perspectives in the Epigenetics of Climate Change-Induced Forests Decline

**DOI:** 10.3389/fpls.2021.797958

**Published:** 2022-01-04

**Authors:** Isabel García-García, Belén Méndez-Cea, David Martín-Gálvez, José Ignacio Seco, Francisco Javier Gallego, Juan Carlos Linares

**Affiliations:** ^1^Departamento de Genética, Fisiología y Microbiología, UD Genética, Facultad de CC Biológicas, Universidad Complutense de Madrid, Madrid, Spain; ^2^Departamento de Biodiversidad, Ecología y Evolución, UD Zoología, Facultad de CC Biológicas, Universidad Complutense de Madrid, Madrid, Spain; ^3^Departamento de Sistemas Físicos, Químicos y Naturales, Universidad Pablo de Olavide, Seville, Spain

**Keywords:** epigenetics, climate change, forest tree species, abiotic stress, methylation

## Abstract

Forest tree species are highly vulnerable to the effects of climate change. As sessile organisms with long generation times, their adaptation to a local changing environment may rely on epigenetic modifications when allele frequencies are not able to shift fast enough. However, the current lack of knowledge on this field is remarkable, due to many challenges that researchers face when studying this issue. Huge genome sizes, absence of reference genomes and annotation, and having to analyze huge amounts of data are among these difficulties, which limit the current ability to understand how climate change drives tree species epigenetic modifications. In spite of this challenging framework, some insights on the relationships among climate change-induced stress and epigenomics are coming. Advances in DNA sequencing technologies and an increasing number of studies dealing with this topic must boost our knowledge on tree adaptive capacity to changing environmental conditions. Here, we discuss challenges and perspectives in the epigenetics of climate change-induced forests decline, aiming to provide a general overview of the state of the art.

## Climate Change-Induced Tree Dieback and Forests Decline

Rising temperatures are exacerbating drought stress for trees at global scales, causing events of decline in several species (usually known as forest dieback; Allen et al., 2010). Climatic projections indicate that mean global temperatures are expected to rise at least 1.5°C until 2100 in response to anthropogenic emissions (IPCC, 2018). Such projections of warming indicate the duration and severity of droughts are expected to amplify, probably leading to widespread forest dieback and tree mortality, affecting tree species currently not assumed to be prone to drought stress, for instance in tropical or boreal forests, or already affected by drought stress, such as those of the Mediterranean forests (Allen et al., 2010). Even though several mechanisms have been described as physiological drivers of dieback, including hydraulic failure and carbon starvation (McDowell et al., 2016; Choat et al., 2018), we lack forecasting tools with sufficient predictive power, since the likelihood of drought-induced tree dieback and mortality is not straightforward (Trugman et al., 2021). This mismatch enhances the need to understand the processes that modulate contrasting adaptive capacity among trees within a given declining forest in order to be able to provide, for instance, adaptive management strategies. Epigenetic processes are known to be able to modulate the patterns of gene expression in plants (Rapp and Wendel, 2005), some being involved in physiological and morphological responses to drought (e. g., Zheng et al., 2013; Liang et al., 2014; Chwialkowska et al., 2016). As drought-induced tree mortality has far-reaching consequences that affect a wide-range of fields, from environmental conservation to climate change mitigation efforts, and it is expected to be more frequent under a climate-change scenario, we need to improve our knowledge about the degree to which epigenetic mechanisms are linked to mortality predictions (e. g., Sow et al., 2018; Amaral et al., 2020; González-Benito et al., 2020).

Trees are capable of coping with short drought periods by avoiding water loss. Thereby, several morphological and physiological adaptations have been developed by forest tree species, for instance variations in tracheids size (Carvalho et al., 2015), reduced leaf area, embedded stomatas, and quick stomata close (Sánchez-Salguero et al., 2015; Kijowska-Oberc et al., 2020). However, long-lasting and severe drought periods are predicted to occur more often, so these strategies might not be enough for their survival (IPCC, 2018).

Tree growth can be used as a marker to study forest tree response to environment alterations, as it is affected by drought stress. Dendrochronology studies have evinced growth decline during drought stress periods, which eventually leads to forest dieback (Camarero et al., 2015; Cailleret et al., 2017). Thus far, drought-induced forests dieback has been observed in several conifer species found in the Mediterranean region, like *Abies pinsapo* Boiss. (Linares et al., 2010), *Abies alba* Mill. (Macías et al., 2006; Camarero et al., 2011, 2015), and *Cedrus atlantica* (Endl.) Manetti ex Carrière (Linares et al., 2011). The Mediterranean basin is considered a climate change hotspot, being one of the most vulnerable areas in the world. As this region was an important refuge in the last glaciation, a large number of endemic and relict species can be found there. Hence, variations in the environment could result in death of these drought-sensitive species which will trigger a huge loss of biodiversity.

In addition, large-distributed forest tree species, like *Pinus sylvestris* L., often live under different environment conditions (Bose et al., 2020), so individuals must show variations that allow them to cope with those circumstances (Camarero et al., 2015; DeSoto et al., 2020). Studying not only endemic species but also largely distributed ones and identifying these differences could be key to understand why some individuals survive while others die (Cailleret et al., 2017).

Surprisingly, there is a lack of data about epigenomic changes in natural populations in spite of their potential role in adaptation to a rapidly changing environment. The most common studies compare individuals under drought conditions with regularly watered individuals, grown in greenhouses (e.g., Gourcilleau et al., 2010; Xu et al., 2018; Li et al., 2020). While this approach allows to accurately control the factors affecting the plants and makes the experiments replicable, it may lack realism, especially when working with long-lived perennial plants due to restriction of their rooting volume, which hinders the extrapolation of the results to natural populations (Gibson et al., 2004). Therefore, studying epigenetic variation in natural populations would be of great interest to understand how trees respond to climate change in the nature.

On summary, as said before, growth of forest tree species is usually slow and the upcoming climate variations will likely be faster than the speed of allele frequencies shift of forest trees, which emphasizes a likely role of epigenetic modifications as a more rapid way of adaptation.

## Epigenetic Mechanisms Under a Climate Changing Scenario

Forest trees are sessile organisms which must cope with environment variations since they are not able to migrate to ensure their survival. Hence, they possess other abilities to compensate for their lack of mobility, such as phenotypic plasticity (Petit and Hampe, 2006). Phenotypic plasticity is the capacity to develop phenotypic modifications in response to multiple environmental factors, increasing the probability of surviving (West-Eberhard, 2008). This mechanism is the result of molecular modifications that trigger changes in gene expression. One of the most important processes involved in phenotypic plasticity is epigenetic regulation, which produces changes in genes expression without modifying the DNA sequence (Iwasaki and Paszkowski, 2014). In fact, phenotypic plasticity is one of the main biological processes that allows trees to respond to rapid variations, like climate change (Alberto et al., 2013). Climate change exacerbates drought stress for trees at global scales, causing stand-level growth decline and episodes of mortality (Allen et al., 2010; Anderegg et al., 2013). As drought-induced forests decline and mortality are reported at an unprecedented rate (Jump and Peñuelas, 2005), a particularly urgent scientific challenge is to understand epigenetic mechanics linking an environmental cue, here increasing drought stress, received by adult trees and its resulting effects on the phenotype of coming generations (Alberto et al., 2013).

In particular, conifers, a group of forest tree species which include ecologically and economically important genera like *Pinus*, *Picea*, and *Abies*, seem to be particularly drought-sensitive (McDowell et al., 2016; DeSoto et al., 2020) and prone to a high rate of epigenetic regulation due to their large genome sizes with high amounts of non-coding DNA (Yakovlev et al., 2012). The study of growth patterns in declining conifer forests suggests a lower capacity of resilience in stressed/dying trees. In fact, trees that died after drought were less resilient after previous droughts, relative to surviving conspecific individuals. Specifically, drought-related mortality risk in conifers has been associated with a low capacity to recover after drought (DeSoto et al., 2020), whereas the epigenetic mechanisms involved on this recovery mechanisms remain largely unknown. Here, we hypothesize that epigenetic mechanisms are likely related to the fate of trees that survive after drought, while some conspecifics died.

Three relevant epigenetic mechanisms have been described: DNA methylation, histone modifications and non-coding RNAs (ncRNAs). Of these, DNA methylation is the most studied one in plants, both model and non-model (Zhang et al., 2018). Two main reasons are responsible of this fact. In one hand, DNA methylation has been reported to be implicated in significant biological processes, like silencing of Transposable Elements (TEs), gene transcription regulation, plant development, and response to biotic and abiotic stress (Zhang et al., 2018). On the other hand, several genomic tools have been developed to easily study at a global level the methylations present in the DNA. Therefore, in this review we will pay special attention to this epigenetic mark.

Regarding histone modifications, these epigenetic marks are involved in chromatin compaction, playing a role in determining its state, which can be decondensed (euchromatin) or condensed (heterochromatin). Euchromatin, also known as open chromatin, is characterized by the presence of a high number of genes, which are usually susceptible to transcription as they are accessible to transcription machinery, while heterochromatin, also known as closed chromatin, is characterized by repetitive and silenced sequences (Amaral et al., 2020). Acetylations, methylations, phosphorilations, and ubiquitylations are some of the panoply of modifications that histones can be subjected to Bannister and Kouzarides (2011), creating patterns that are associated to different transcriptional states. Unfortunately, these modifications are numerous and challenging to analyze in trees as standard chromatin immunoprecipitation (ChIP) procedures are hard to apply to woody cells and tissues (Li et al., 2014). Thus, very few studies regarding histone modifications in trees have been published to date.

Non-coding RNAs are also rather relevant as an epigenomic mechanism, as they are mobile within and between cells and are not lost during cell divisions, unlike the majority DNA methylations and histone modifications (Yakovlev et al., 2012). They are classified by their length: if they have more than 200 nucleotides, they are called as long ncRNAs (lncRNAs) whereas if they have less than 200 nucleotides, they are called short ncRNAs. These short ncRNAs include micro RNAs (miRNAs), short interfering RNAs (siRNAs), and Piwi-interacting RNAs (piRNAs), and they are involved in genomic imprinting, X-chromosome inactivation, repression of gene transcription, and transposon repression (Wei et al., 2016). Therefore, given the elevated content of TEs in conifer genomes, ncRNAs stand as a major epigenetic mark that should be studied in depth.

Lastly, DNA methylation positions itself as a highly relevant epigenetic mark in conifers due to their large content in TEs and its role in silencing them. In addition, there are numerous studies that demonstrate its participation in the control of gene expression in a large number of traits, in plants in general and in conifers in particular, which will be addressed later. DNA methylation is a covalent change which consists in the addition of a methyl group to a cytosine residue, in most cases to its fifth carbon position, and can be, in some cases, inherited by the offspring. In plants, DNA methylation happens not only in CpG sites but also in CHG and CHH contexts, where H can be A, T, or C (Law and Jacobsen, 2010). CpG sites are the most frequent mark in/near protein-coding gene sequences (Ashapkin et al., 2020) especially of those with constitutive expression. Although this methylation is known to affect gene expression, its role is still unclear, as it can lead to both silencing and increased expression (Zemach and Ziberman, 2010). Regarding CHG and CHH sites, different studies have highlighted that they are mainly present in repetitive sequences and TEs, both abundant in tree genomes, and their methylation usually reduces or even blocks DNA transcription, allowing the maintenance of the genome integrity (Zemach et al., 2010a; Bewick and Schmitz, 2017).

It has been proved that DNA methylation is responsible of phenotypic variation in plants (Martin et al., 2009; Mougninot et al., 2021; Zhao et al., 2021). Considering that different types of stress, such as abiotic stress, can trigger methylation, eventually leading to variation of gene expression, this field of research has become a useful tool for understanding how tree species deal with the environment variations. Several studies have highlighted that epigenetics seems to act like a memory process to respond to different environmental stresses, which is called “stress memory” (Kinoshita and Seki, 2014). Thus, a new question arises: could it be possible to inherit this stress-induced DNA methylations? Most of the time, epigenetic marks are erased to start over in the progeny, however it is possible for this reset to be incomplete, allowing some marks to pass on to the offspring. Moreover, it is known that some germline cells come from somatic cells in plants, so epigenetic marks that appear in those cells could be passed on to the offspring. Hence, epigenetic marks can be transmitted to the offspring by meiosis in some cases. Studies carried out with *Arabidopsis thaliana* L. (Cervera et al., 2002; Boyko et al., 2010; Lang-Mladek et al., 2010) or *Linaria vulgaris* Mill. (Cubas et al., 1999) evidenced epigenetic modifications related to environment variations can be inherited. However, the mechanisms involved in this process are not well understood to date.

All in all, epigenetic modifications could be a rapid way to achieve short-term adaptations, since epigenetic marks bring new phenotypes which might affect fitness and, thus, the underlying evolutionary processes. Particularly, it could be key for forest trees survival, as they are sessile long-lived organism with long generation times, so the possible beneficial effects of natural selection on them are limited and slow and will soon be surpassed by the effects of climate change.

## Techniques for Epigenomic Analysis

Over the years, different techniques have been developed to study DNA methylation. Their first requirement is high-quality DNA, which highlights the importance of selecting an appropriated method for sample conservations during and after each collection, as well as an extraction method as efficient as possible for each type of samples. Once the DNA is obtained, there is a wide variety of methods than can be used to analyze its methylation patters (Table 1). The most remarkable ones are shown below.

**TABLE 1:** Summary of the advantages and weaknesses of the most relevant DNA methylation techniques and their suitability for species without reference genomes.

Technique	Advantages	Weaknesses	Suitable for species without reference genome
MeDIP-seq	Low initial amount of DNA. Genome-wide scale.	No single base resolution. It does not discriminate methylation contexts. Relative levels of methylation.	No
MRE-seq	Reduction of the complexity of genomes. Single-base resolution.	Low coverage of the genome. Relative levels of methylation.	No
MSAP-seq	Reduction of the complexity of genomes. Useful with large-size genomes.	Low coverage of the genome. Loss of information of CHH and CHG contexts.	Yes
WGBS	It uses entire genomes. Single-base resolution. All contexts are represented. Absolute levels of DNA methylation.	Not suitable for large-size genomes.	No
RRBS	Reduction of the complexity of genomes. Single-base resolution. All contexts are represented.	Low coverage of the genome.	Yes

At the beginning, methods based on antibodies affinity to methylcytosine residues were used. One of them is the well-known methyl-DNA immunoprecipitation (MeDIP) technique, which captures the methylated DNA fragments with an antibody against methylated cytosines (Thu et al., 2009). Then a new approach of this technique, called MeDIP-seq, was developed (Zhao et al., 2014). The novelty of this method consisted in a final sequencing step based in next-generation sequencing (NGS). Both techniques require a previous DNA sonication step to obtain random DNA fragments. MeDIP-seq is frequently used in combination with MRE-seq (methylation sensitive restriction enzyme sequencing) (Maunakea et al., 2010). MRE-seq consists of a DNA fragmentation using several restriction enzymes which are methylation sensitive followed by a final sequencing step. Although MeDIP-seq and MRE-seq can be used independently, their combination can increase the accuracy of DNA-methylation studies (Li et al., 2015).

Subsequently, an improvement based on NGS allowed to modify the methyl-sensitive amplified polymorphism (MSAP) technique, giving rise to MSAP-seq. Originally, the MSAP technique appeared as a modification of the amplified fragment length polymorphism method (AFLP). This technique was described by Reyna-López et al. (1997) in fungi and then Xiong et al. (1999) adapted it to work with plants. MSAP starts with a genome fragmentation using restriction enzymes with differential sensitivity to cytosine methylation. *Msp*I and *Hpa*II are one of the enzyme isoschizomer pairs used in MSAP. Both recognize the same target 5’-CCpGG-3’, but *Hpa*II will not cleave if the inner cytosine residue is methylated. Hence, this pair is used to identify differences in methylation marks between DNA samples in CpG contexts mainly. Following the cleavage step, a PCR amplification is carried out with the aim to obtain an overrepresentation of previously obtained fragments. Then, amplicons are separated and visualized via gel electrophoresis. MSAP-seq replaces this last sept by a sequencing one. This method was first described by Chwialkowska et al. (2016) in barley and was later improved to work with large genome species (Chwialkowska et al., 2017). It allows studies in non-model species with lack of a reference genome. However, restriction enzymes seem to cut less efficiently in CHH and CHG contexts than in CpG islands, which may lead to mistakes in methylation calling, which in turn might imply a loss in methylated states. This could be a remarkable weakness of this technique considering that the larger genome size is, the greater amount of cytosine methylations there is in the other contexts.

Finally, new approaches based on sodium bisulfite treatments have been developed to study DNA methylation, since bisulfite sequencing was developed in 1992 (Frommer et al., 1992). Bisulfite methods are useful to study genome-wide methylation and provide high single-base resolution. These techniques convert unmethylated cytosines to uracils, as methylated cytosines are protected against the deamination effect produced by the bisulfite treatment. After that, uracils are turned into thymines in a PCR amplification. Then, these changes can be easily identified by sequencing. To date, there are two bisulfite techniques: whole genome bisulfite sequencing (WGBS) and reduced representation bisulfite sequencing (RRBS), which is subdivided in three protocols based on RAD-seq (Baird et al., 2008) and genotyping by sequencing (GBS; Elshire et al., 2011) techniques: epiRADseq (Schield et al., 2016), bsRADseq (Trucchi et al., 2016), and epiGBS (van Gurp et al., 2016).

WGBS works with the entire genome, while the RRBS method targets specific regions. In RRBS methylation sensitive restriction enzymes are used to fragment the whole genome, and then DNA fragments are filtered by size. The characteristics of WGBS make it suitable for studies with model organisms with short length genome size and for species with a reference genome (Paun et al., 2019). On the other hand, the genome fragmentation carried out with RRBS methods constitutes a useful tool to reduce the complexity of genomes (Davey et al., 2011), which allows to work with large-size genomes, with or without a reference genome.

Although this review is focused on DNA methylation, here is a brief description of other techniques used to study the remaining epigenetic marks: histone modifications and ncRNAs.

One of the most common techniques to study *in vivo* histone modifications is chromatin immunoprecipitation (ChIP). First, samples are treated with formaldehyde to cross-link the chromatin interactions (protein–protein and protein–DNA). Then, small fragments are obtained by sonication or nuclease digestion. Next, specific antibodies are used to retain the DNA-histone modification complex of interest. Finally, different methods can be carried out to determine these regions such as PCR, real-time PCR, Southern blot, and Western blot. The development of NGS techniques has led to the addition of a final sequencing step to the ChIP protocol, creating the technique known as ChIP-seq. However, the presence of polysaccharide and phenolic compounds in plants may difficult these experiments, that could turn them into a challenge in some cases (Muhammad et al., 2020).

The most common technique used to study ncRNA sequences is RNA-seq (Wang et al., 2009). Then, sequencing data can be combined with RT-qPCRs to determine expression levels, or with fluorescence *in situ* hybridization (FISH) experiments to localize the ncRNAs in a cellular context, depending on the aim of the study. An *in silico* approach consists in looking for previously described or computationally predicted ncRNAs in the genomic region under study. Several databases of ncRNAs sequences are available online, such as CANTATAdb 2.0^1^ (Szcześniak et al., 2015) which has over 200,000 lncRNAs from 39 plant species from which 2 are trees. As usual, forest trees are poorly represented in this kind of databases, mainly due to the lack of reference genomes.

## In Search of a Reference

Forest trees span a huge range of genome sizes, including species with less than 1 Gb, such as the *Populus* genus, to the conifer giga-genomes, whose size varies from ∼6.5 to ∼37 Gb, being ∼15 Gb the most common 1C-value (Ahuja and Neale, 2005). Compared to the ∼0.6 Gb average of most angiosperms (Dodsworth et al., 2015), conifer genomes are remarkably large and, therefore, constitute a challenging work scenario for genomic and epigenomic studies. In addition, a great number of experimental techniques and bioinformatic pipelines are designed work with the 3.3 Gb-long human genome, making it difficult to adapt them to genomes which are on average five times bigger. For instance, techniques aimed at sequencing complete genomes, such as WGBS, might not be suitable for giga-genomes as optimal depth and coverage values will be hard to achieve.

To complicate matters further, approximately 75% of conifer genomes consists of highly repetitive DNA, mainly represented by non-coding DNA, such as mobile elements, along with rDNA repeat units (Ahuja and Neale, 2005). In addition, gene duplications have created large gene families, which are not necessarily found in certain regions but dispersed across the genomes (Kinlaw and Neale, 1997).

On the other hand, luckily, polyploidy is not a common phenomenon in conifers, and it has only been reported in the Cupressaceae family (Ahuja, 2005). All in all, however, the global genetic and, thus, epigenetic landscapes of these tree species do not provide a research-friendly environment.

While many plant genomes are being sequenced and assembled every year due to the improvement of next generation sequencing techniques and their decrease in cost (Mackay et al., 2012), few conifer reference genomes are available. The first gymnosperm giga-genome ever published was that of *Picea abies* (L.) H. Karst. (Nystedt et al., 2013). Since then, six more conifer genomes have been sequenced: *Pinus taeda* L. (Neale et al., 2014), *Picea glauca* (Moench) Voss (Warren et al., 2015), *Pinus lambertiana* Douglas (Stevens et al., 2016), *Pseudotsuga menziesii* (Mirb.) Franco (Neale et al., 2017), *Larix sibirica* Ledeb. (Kuzmin et al., 2019), and *Abies alba* Mill. (Mosca et al., 2019). The N50 statistic is used as an indicator for genome assembly quality. It is defined as the length of the contig that reaches 50% of the total genome length, being the contigs sorted by length in descending order. Therefore, the higher this value is, the better the assembly is, as the median contig size will be longer. The total length of the previously mentioned genomes ranges from ∼12 to ∼32 Gb, with N50 scaffold sizes between ∼5 to ∼2,500 Kb, which shows that further refinement is still needed. Nevertheless, over 45,000 genes have been successfully annotated in each of the cited examples.

The recent upsurge in popularity and number of users of third generation sequencing technologies, also known as long-read sequencing, could provide a substantial boost to the improvement of the quality of the existing reference genomes. Long reads, alone or in combination with short reads, have a high potential for assembling and re-assembling genomes (Amarasinghe et al., 2020). The reads obtained from third generation sequencing methods, such as PacBio single-molecule real-time (SMRT) and Oxford Nanopore, constitute a less fragmented representation of large genomes, facilitating their assembly in longer scaffolds. For instance, the quality of the reference genome of barley (*Hordeum vulgare* L.), which is not a forest tree but whose genome shares some characteristics with the conifer ones, such as being highly repetitive and ∼5 Gb-long, was superior for long-read assemblies than for the one based on short reads, obtaining higher contig N50 values (Mascher et al., 2021).

On the other hand, sequencing of transcriptomes is more extensive in conifers, due to their notably reduced size compared to whole genomes, which reduces costs and increases the probability of capturing the majority of the sequences. The One Thousand Plant Transcriptomes Initiative database^2^ collects over 1,300 transcriptomes of green plants, of which 76 belong to 72 different conifer species, coming from diverse tissue samples, including leaves, branches, and young shoots. Hardwood Genomics Project^3^ and TreeGenes^4^ are two other examples of databases specialized in trees that include transcriptomes as well as genomes, annotations and genetic markers.

However, the information coming merely from transcriptome sequencing is clearly insufficient to address the study of epigenetic mechanisms in conifers. Some of the most common epigenomic methodologies, such as identification of differentially methylated regions (DMRs) and differentially methylated promoters (DMPs), need the information of the sequences surrounding the coding regions. Then, the transcripts identified in transcriptomic experiments are useless without a reference to provide their genomic context.

Regarding complete methylomes, it has been described that CpG and CHG methylation is positively correlated with genome size (Ausin et al., 2016; Takuno et al., 2016), and high methylation levels are also known to be characteristic of genomes with a high content of transposable elements, as they constitute the main target of DNA methylation (Baduel and Colot, 2021). The lack of methylation maps of conifers is therefore not surprising, given the vast amount of data that needs to be analyzed to create them. Withal, the Norway spruce (*P. abies*) DNA methylation map was successfully obtained (Ausin et al., 2016). As previously mentioned, the genome of this species is fully sequenced, which highlights the importance of having reference genomes in order to be able to conduct this type of high throughput epigenetic studies.

## Bioinformatic Tools

Bioinformatic pipelines for the analysis of DNA methylation data obtained after bisulfite treatments consist of four main steps: quality check and read trimming, read mapping, methylation calling, and identification of differentially methylated regions (Wreczycka et al., 2017).

Quality check and trimming of raw reads can be performed with well-known software, including FastQC (Andrews, 2010), and Trimmomatic (Bolger et al., 2014) and Cutadapt (Martin, 2011), respectively. Generally, these steps include removing adapter sequences, filtering out low-quality reads and trimming reads of low-quality bases. Specific bisulfite-sequencing tools are also available, such as BSeQC (Lin et al., 2013), which evaluates quality and automatically trims technical biases that may interfere in accurate methylation calling. For example, it can detect 5’ bisulfite conversion failure and remove the reads affected by this bias, which causes artificially high methylation rates at that end of the reads.

Bisulfite conversion rates should also be checked, especially if non-CG methylation levels are the focus of the study. Non-CG context, as previously mentioned, play an important role in silencing repetitive sequences and TEs, which are highly abundant in conifer genomes, so their study could be of great interest. In particular, CHH contexts can be quite problematic due to their low methylation levels (Takuno et al., 2016), which require a high bisulfite conversion rate to ensure that they are not underestimated. Thus, a spike-in control, such as unmethylated lambda phage DNA, could be useful to check that the bisulfite conversion is working at optimal levels.

Read mapping is a particularly challenging step. After bisulfite treatment, unmethylated cytosines are converted to uracils and then to thymines in the following PCR amplification, while methylated cytosines are not affected. This creates a need for special reference genomes, modified to take into consideration these nucleotide changes. In addition to the increased mapping searching sequence, asymmetric cytosine to thymine alignments and multiple CpG heterogeneous methylation must be taken into consideration by the mapping software (Xi and Li, 2009). Nonetheless, several bisulfite mapping tools have been developed, such as BSMAP (Xi and Li, 2009) and Bismark (Krueger and Andrews, 2011). Recently, Grehl et al. (2020) compared the efficiency and impact of the mapping results on the identification of DMRs of eight mappers and concluded that these two are the best options, as they showed the highest precision with the shortest run time and lowest memory requirements, respectively.

Both tools also provide methylation level files showing methylation calling status for each cytosine position. The coverage files produced by the Bismark methylation extractor function can be directly used as inputs for DMR-finding programs, such as the popular edgeR package (Robinson et al., 2010), or can easily be edited to fit other tools’ input requirements, such as DSS (Park and Wu, 2016) and swDMR (Wang et al., 2015).

In addition to these tools that allow users to perform each step of the analysis separately, others combine all the procedures in one pipeline. Bicycle (Graña et al., 2017) and SMAP (Gao et al., 2015) are some of the recommended all-in-one software for WGBS and RRBS data analysis, respectively (Rauluseviciute et al., 2019).

Despite these apparently wide variety of tool choices, there are several issues to take into consideration when it comes to analyzing high-throughput bisulfite sequencing data. First, the need of a good quality reference genome to perform the read mapping step, which is not a frequent situation, as previously explained. Besides, some tools, like swDMR, require an annotation file as input, whose quality relies on the genome quality itself. For instance, if a DMR falls in a region that is not described, likely due to genome fragmentation caused by large number of scaffolds or small scaffold sizes, this DMR will provide little information regarding its biological meaning. In addition, the amount of Cs that would need to be analyzed will in all probability be quite high in comparison to other non-conifer species. Therefore, the computational requirements in epigenomic experiments on forest tree species are likely to be very demanding, particularly in terms of RAM memory usage, storage space and run time.

Researchers that choose non-bisulfite-based techniques are likely to face the above-mentioned problems derived from genome size and lack of references, as they also include a next-generation sequencing step. Nonetheless, as in the case of bilsulfite-based methods, there are many bioinformatic tools specifically designed to work with this kind of data. The MeDUSA pipeline (Wilson et al., 2012) and Batman software (Down et al., 2008) are suitable for MeDIP-seq data analysis, while the MSEQER pipeline (Chwialkowska et al., 2017) is designed to work with MSAP-seq data.

## Previous Studies

As it is said above, studies of epigenetic marks in plants are mainly focused on DNA methylation (Kurdyukov and Bullock, 2016). Nevertheless, the role of histone acetylations in root growth and development (Ma et al., 2016) and in photosynthesis (Li et al., 2017) has been described in different species of poplar, as well as that of miRNAs in growth and phosphorus nutrition (Schönberger et al., 2016). It is worth noting that this kind of studies have been carried out on poplars because they are considered a forest tree model organism. As mentioned above, poplar has a small genome (∼0.5 Gb) with a low proportion of repeated sequences. This genome has been fully sequenced and annotated, so its genomic resources are of high quality. Except in this particular case, histone modifications and ncRNAs remain mostly undescribed in conifer species and represent a major challenge to advance in the knowledge of epigenomics in trees.

The first plant methylome published was that of *A. thaliana* L. (Cokus et al., 2008), and it was developed using a large-scale bisulfite sequencing experiment, while the first conifer one, of *P. abies*, did not come until 2016 (Ausin et al., 2016).

Over the years, some studies have focused on changes in DNA methylation during normal plant development (e.g., Bitonti et al., 2002; Zemach et al., 2010b; Perrin et al., 2020) and heteroblastic development (Hasbún et al., 2016). Others are about phenotypic plasticity and stress memory (e.g., Herrera and Bazaga, 2013; Le Gac et al., 2018). What it is common to the vast majority of them is the use of model species, like *A. thaliana* (e.g., Korotko et al., 2021; Markus et al., 2021), and important commercial species such as crops (e.g., Feng et al., 2010; Wang et al., 2010; Karan et al., 2012; Bhanu et al., 2020; Merce et al., 2020) or fruit trees (e.g., Avramidou et al., 2015; Badad et al., 2021; Rothkegel et al., 2021).

The knowledge about forest tree epigenetics is scarce. Nevertheless, some studies related with epigenetic memory during embryogenesis have been developed on forest trees of species such as *P. abies* (Kvaalen and Johnsen, 2008; Yakovlev et al., 2014, 2016), *P. glauca* (Webber et al., 2005), or *P. sylvestris* (Dormling and Johnsen, 1992), and more studies are coming (e.g., Lafon-Placette et al., 2018; Vanden Broeck et al., 2018).

Furthermore, studies have generally focused on the involvement of DNA methylation in abiotic stresses response and adaptation in forest trees and other plants (Table 2). Modifications in response to water have been found in the methylome of *Populus* species (Gourcilleau et al., 2010; Raj et al., 2011), as well as variations in the methylation pattern of *Pinus radiata* D. Don. in response to heat stress (Lamelas et al., 2020). Again, it is remarkable the huge amount of poplar (*Populus* spp.) studies which have been carried out over the years, thanks to the availability of a quality reference genome and its wide phenotypic variation.

**TABLE 2:** Brief description of some DNA methylation studies carried out with the main objective of studying plant species response to several abiotic stresses.

Plant species	Technique	Objective	Reference genome	References
*Bruguiera gymnorhiza* (L.) Lamk.	WGBS	Salt stress response	No	Miryeganeh et al., 2021
*Ilex aquifolium* L.	MSAP	Epigenetic variability	No	Herrera and Bazaga, 2013
*Medicago sativa* L. and *Medicago* spp	MSAP	Salt stress response	Yes	Al-Lawati et al., 2016
*Morus alba* L.	WGBS	Drought stress response	Yes	Li et al., 2020
*Quercus ilex* L.	MSAP	Drought stress response	No	Rico et al., 2014
*Quercus lobata* Née	RRBS	Environment response	Yes	Gugger et al., 2016
*Eucalyptus globulus* Labill.	MeDIP-seq, MRE-seq	Heteroblastic development	Yes	Hasbún et al., 2016
*Populus simonii* Carr.	MSAP	Various abiotic stresses	Yes	Song et al., 2016
*Populus trichocarpa* Hook.	WGBS	Drought stress response	Yes	Liang et al., 2014
*Populus euphratica* Oliv.	WGBS	Salt stress response	No	Su et al., 2018
*Populus tremula* L. *x Populus alba* L.	RNAi, HPLC, WGBS	Drought stress response	No	Sow et al., 2021
*Pinus pinea* L.	MSAP	Epigenetic variability	No	Sáez-Laguna et al., 2014

Studies in natural populations are of particular importance to understand how epigenetic modifications can contribute to adaptation to the new climatic conditions caused by climate change. One of these studies was carried out with *P. sylvestris* (Alakärppä et al., 2018), which is a widely distributed conifer, likely to show variations in its epigenetic marks as it can be found under quite different environmental scenarios, and a link between local adaptation and environment condition, and modifications in DNA methylation has been suggested. Another work with samples of natural populations of *Populus simonii* Carr. (Ci et al., 2016) was conducted to analyze population epigenetic structure, which showed a substructure population associated to different methylation patterns.

In the literature, several reviews about epigenetics in plants and forest trees can be found (Paun et al., 2019; Amaral et al., 2020; Ashapkin et al., 2020; Zhao et al., 2021). Most of them are focused on the epigenetic response to specific abiotic stresses, such as drought, salt, or heavy metals, and they are useful as an introduction to this challenging field. On top of that, this review should prove valuable to researchers who wish to design an experiment to study methylation, as it covers the whole process: from choosing an appropriate technique to performing the bioinformatic analysis of the data.

To sum up, epigenomics open a research field to identify some of the processes related with phenotypic plasticity and its study will bring us a better understanding of adaptive forest tree response to a changing environment. Fortunately, substantial efforts are being made to improve DNA methylation techniques to make them suitable and cost-effective for non-model species, specifically with trees or species with large genomes (e.g., Chwialkowska et al., 2017; Pereira et al., 2020; Werner et al., 2020).

## Conclusion

It is expected that the growth rates of forest tree species become on average slow, while the upcoming climate change trends become faster than the rate of allele frequencies shift of forest trees. This emphasizes the putative role of epigenetic modifications as a fast adaptive mechanism to cope with current warmer and drier climate scenario.

Phenotypic plasticity is the principal strategy to compensate for trees’ lack of ability to move when it comes to dealing with rapid environmental variations, such as ongoing climate change. It is based on modifications of genetic regulation which depend on epigenetic mechanisms such as histone modifications, DNA methylation and non-coding RNAs. Despite of the importance of these processes, epigenomic studies in forest tree species are challenging and scarce, mainly due to their complex genomes and lack of references.

Throughout this review, we have gathered enough information to give a general overview about the key points for methylation experiments design when dealing with forest trees. We have also highlighted opportunities for future advances in this framework and the relevance of dedicating efforts to improve the techniques for epigenomic analysis and the bioinformatic tools to make them suitable for large-size genomes.

## Author Contributions

IG-G and BM-C wrote the manuscript. All authors discussed the results and contributed to the final manuscript.

## Conflict of Interest

The authors declare that the research was conducted in the absence of any commercial or financial relationships that could be construed as a potential conflict of interest.

## Publisher’s Note

All claims expressed in this article are solely those of the authors and do not necessarily represent those of their affiliated organizations, or those of the publisher, the editors and the reviewers. Any product that may be evaluated in this article, or claim that may be made by its manufacturer, is not guaranteed or endorsed by the publisher.

## Footnotes

1
http://cantata.amu.edu.pl/

2
http://www.onekp.com

3
https://hardwoodgenomics.org/

4
https://treegenesdb.org/

## References

[B1] Ahuja M. R. (2005). Polyploidy in gymnosperms: Revisited. *Silvae Genet.* 54 59–69. 10.1515/sg-2005-0010

[B2] Ahuja M. R. Neale D. B. (2005). Evolution of Genome Size in Conifers. *Silvae Genet.* 54 126–137. 10.1515/sg-2005-0020

[B3] Alakärppä E. Salo H. M. Valledor L. Cañal M. J. Häggman H. Vuosku J. (2018). Natural variation of DNA methylation and gene expression may determine local adaptations of Scots pine populations. *J. Exp. Bot.* 69 5293–5305. 10.1093/jxb/ery292 30113688

[B4] Alberto F. J. Aitken S. N. Alia R. Gonzalez-Martinez S. C. Hanninen H. Kremer A. (2013). Potential for evolutionary responses to climate change evidence from tree populations. *Glob. Change Biol.* 19 1645–1661. 10.1111/gcb.12181 23505261 PMC3664019

[B5] Al-Lawati A. Al-Bahry S. Victor R. Al-Lawati A. H. Yaish M. W. (2016). Salt stress alters DNA methylation levels in alfalfa (*Medicago* spp). *Genet. Mol. Res*. 15 1–16. 10.4238/gmr.15018299 26985924

[B6] Allen C. D. Macalady A. K. Chenchouni H. Bachelet D. McDowell N. Vennetier M. (2010). A global overview of drought and heat-induced tree mortality reveals emerging climate change risks for forests. *For. Ecol. Manage.* 259 660–684. 10.1016/j.foreco.2009.09.001

[B7] Amaral J. Ribeyre Z. Vigneaud J. Sow M. D. Fichot R. Messier C. (2020). Advances and Promises of Epigenetics for Forest Trees. *Forests* 11:976. 10.3390/f11090976

[B8] Amarasinghe S. L. Su S. Dong X. Zappia L. Ritchie M. E. Gouil Q. (2020). Opportunities and challenges in long-read sequencing data analysis. *Genome Biol.* 21:30. 10.1186/s13059-020-1935-5 32033565 PMC7006217

[B9] Anderegg W. R. L. Kane J. M. Anderegg L. D. L. (2013). Consequences of widespread tree mortality triggered by drought and temperature stress. *Nat. Clim. Change* 3 30–36. 10.1038/nclimate1635

[B10] Andrews S. (2010). *FastQC*. *A quality control tool for high throughput sequence data.* Available online at: http://www.bioinformatics.babraham.ac.uk/projects/fastqc

[B11] Ashapkin V. V. Kutueva L. I. Aleksandrushkina N. I. Vanyushin B. F. (2020). Epigenetic mechanisms of plant adaptation to biotic and abiotic stresses. *Internat. J. Mole. Sci.* 21:7457. 10.3390/ijms21207457 33050358 PMC7589735

[B12] Ausin I. Feng S. Yu C. Liu W. Kuo Y. Jacobsen E. L. (2016). DNA methylome of the 20-gigabase Norway spruce genome. *Proc. Natl. Acad. Sci. USA* 113 E8106–E8113. 10.1073/pnas.1618019113 27911846 PMC5167160

[B13] Avramidou E. V. Ganopoulos I. V. Doulis A. G. Tsaftaris A. S. Aravanopoulos F. A. (2015). Beyond population genetics: natural epigenetic variation in wild cherry (*Prunus avium*). *Tree Genet. Genomes* 11:95. 10.1007/s11295-015-0921-7

[B14] Badad O. Lakhssassi N. Zaid N. El Baze A. Zaid Y. Meksem J. (2021). Genome Wide MeDIP-Seq Profiling of Wild and Cultivated Olives Trees Suggests DNA Methylation Fingerprint on the Sensory Quality of Olive Oil. *Plants* 10:1405. 10.3390/plants10071405 34371608 PMC8309279

[B15] Baduel P. Colot V. (2021). The epiallelic potential of transposable elements and its evolutionary significance in plants. *Philos. Trans. R. Soc. Lond. B. Biol. Sci.* 376:20200123. 10.1098/rstb.2020.0123 33866816 PMC8059525

[B16] Baird N. A. Etter P. D. Atwood T. S. Currey M. C. Shiver A. L. Lewis Z. A. (2008). Rapid SNP Discovery and Genetic Mapping Using Sequenced RAD Markers. *PLoS One* 3:e3376. 10.1371/journal.pone.0003376 18852878 PMC2557064

[B17] Bannister A. Kouzarides T. (2011). Regulation of chromatin by histone modifications. *Cell Res.* 21 381–395. 10.1038/cr.2011.22 21321607 PMC3193420

[B18] Bewick A. J. Schmitz R. J. (2017). Gene body DNA methylation in plants. *Curr. Opin. Chem. Biol.* 36 103–110. 10.1016/j.pbi.2016.12.007 28258985 PMC5413422

[B19] Bhanu B. D. Ulaganathan K. Shanker A. K. (2020). Water Stress Responsive Differential Methylation of Organellar Genomes of *Zea mays* Z59. *Am. J. Plant Sci.* 11:1077. 10.4236/ajps.2020.117077

[B20] Bitonti M. B. Cozza R. Chiappetta A. Giannino D. Castiglione M. R. Dewitte W. (2002). Distinct nuclear organization, DNA methylation pattern and cytokinin distribution mark juvenile, juvenile-like and adult vegetative apical meristems in peach (*Prunus persica* (L.) Batsch). *J. Exp. Bot.* 53 1047–1054. 10.1093/jexbot/53.371.1047 11971916

[B21] Bolger A. M. Lohse M. Usadel B. (2014). Trimmomatic: A flexible trimmer for Illumina Sequence Data. *Bioinform.* 30 2114–2120. 10.1093/bioinformatics/btu170 24695404 PMC4103590

[B22] Bose A. K. Gessler A. Bolte A. Bottero A. Buras A. Cailleret M. (2020). Growth and resilience responses of Scots pine to extreme droughts across Europe depend on predrought growth conditions. *Glob. Change Biol.* 26 4521–4537. 10.1111/gcb.15153 32388882 PMC7383776

[B23] Boyko A. Blevins T. Yao Y. Golubov A. Bilichak A. Ilnytskyy Y. (2010). Transgenerational adaptation of *Arabidopsis* to stress requires DNA methylation and the function of Dicer-like proteins. *PLoS One* 5:9514. 10.1371/journal.pone.0009514 PMC2831073 20209086

[B24] Cailleret M. Jansen S. Robert E. M. Desoto L. Aakala T. Antos J. A. (2017). A synthesis of radial growth patterns preceding tree mortality. *Glob. Change Biol.* 23 1675–1690. 10.1111/gcb.13535 27759919

[B25] Camarero J. J. Bigler C. Linares J. C. Gil-Pelegrín E. (2011). Synergistic effects of past historical logging and drought on the decline of Pyrenean silver fir forests. *For. Ecol. Manag.* 262 759–769. 10.1016/j.foreco.2011.05.009

[B26] Camarero J. J. Gazol A. Sangüesa-Barreda G. Oliva J. Vicente-Serrano S. M. Gibson D. (2015). To die or not to die: early warnings of tree dieback in response to a severe drought. *J. Ecol.* 103 44–57. 10.1111/1365-2745.12295

[B27] Carvalho A. Nabais C. Vieira J. Rossi S. Campelo F. (2015). Plastic Response of Tracheids in Pinus pinaster in a Water-Limited Environment: Adjusting Lumen Size instead of Wall Thickness. *PLoS One* 10:e0136305. 10.1371/journal.pone.0136305 26305893 PMC4549277

[B28] Cervera M. T. Ruiz-García L. Martínez-Zapater J. M. (2002). Analysis of DNA methylation in *Arabidopsis thaliana* based on methylation-sensitive AFLP markers. *Mol. Genet. Genomics* 268 543–552. 10.1007/s00438-002-0772-4 12471452

[B29] Choat B. Brodribb T. J. Brodersen C. R. Duursma R. A. Lopez R. Medlyn B. E. (2018). Triggers of tree mortality under drought. *Nature* 558 531–539. 10.1038/s41586-018-0240-x 29950621

[B30] Chwialkowska K. Korotko U. Kosinska J. Szarejko I. Kwasniewski M. (2017). Methylation Sensitive Amplification Polymorphism Sequencing (MSAP-Seq) - A Method for High-Throughput Analysis of Differentially Methylated CCGG Sites in Plants with Large Genomes. *Front. Plant Sci*. 8:2056. 10.3389/fpls.2017.02056 29250096 PMC5714927

[B31] Chwialkowska K. Nowakowska U. Mroziewicz A. Szarejko I. Kwasniewski M. (2016). Water-deficiency conditions differently modulate the methylome of roots and leaves in barley (*Hordeum vulgare* L.). *J. Exp. Bot.* 67 1109–1121. 10.1093/jxb/erv552 26739862 PMC4753852

[B32] Ci D. Song Y. Du Q. Tian M. Han S. Zhang D. (2016). Variation in genomic methylation in natural populations of Populus simonii is associated with leaf shape and photosynthetic traits. *J. Exp. Bot.* 67 723–737. 10.1093/jxb/erv485 26552881

[B33] Cokus S. J. Feng S. Zhang X. Chen Z. Merriman B. Haudenschild C. D. (2008). Shotgun bisulphite sequencing of the *Arabidopsis* genome reveals DNA methylation patterning. *Nature* 452 215–219. 10.1038/nature06745 18278030 PMC2377394

[B34] Cubas P. Vincent C. Coen E. (1999). An epigenetic mutation responsible for natural variation in floral symmetry. *Nature* 401 157–161. 10.1038/43657 10490023

[B35] Davey J. W. Hohenlohe P. A. Etter P. D. Boone J. Q. Catchen J. M. Blaxter M. L. (2011). Genome-wide genetic marker discovery and genotyping using next-generation sequencing. *Nat. Rev. Genet.* 12 499–510. 10.1038/nrg3012 21681211

[B36] DeSoto L. Cailleret M. Sterck F. Jansen S. Kramer K. Robert E. M. R. (2020). Low growth resilience to drought is related to future mortality risk in trees. *Nat. Comm.* 11:545. 10.1038/s41467-020-14300-5 31992718 PMC6987235

[B37] Dodsworth S. Leitch A. R. Leitch I. J. (2015). Genome size diversity in angiosperms and its influence on gene space. *Curr. Opin. Genet. Dev.* 35 73–78. 10.1016/j.gde.2015.10.006 26605684

[B38] Dormling I. Johnsen Ø (1992). Effects of the parental environment on full- sib families of *Pinus sylvestris*. *Can. J. For. Res.* 22 88–100. 10.1139/x92-013

[B39] Down T. A. Rakyan V. K. Turner D. J. Flicek P. Li H. Kulesha E. (2008). A Bayesian deconvolution strategy for immunoprecipitation-based DNA methylome analysis. *Nat. Biotechnol.* 26 779–785. 10.1038/nbt1414 18612301 PMC2644410

[B40] Elshire R. J. Glaubitz J. C. Sun Q. Poland J. A. Kawamoto K. Buckler E. S. (2011). A Robust, Simple Genotyping-by-Sequencing (GBS) Approach for High Diversity Species. *PLoS One* 6:e19379. 10.1371/journal.pone.0019379 21573248 PMC3087801

[B41] Feng S. Cokus S. J. Zhang X. Chen P. Y. Bostick M. Goll M. G. (2010). Conservation and divergence of methylation patterning in plants and animals. *PNAS* 107 8689–8694. 10.1073/pnas.1002720107 20395551 PMC2889301

[B42] Frommer M. McDonald L. E. Millar D. S. Collis C. M. Watt F. Grigg G. W. (1992). A genomic sequencing protocol that yields a positive display of 5-methylcytosine residues in individual DNA strands. *PNAS* 89 1827–1831. 10.1073/pnas.89.5.1827 1542678 PMC48546

[B43] Gao S. Zou D. Mao L. Zhou Q. Jia W. Huang Y. (2015). SMAP: a streamlined methylation analysis pipeline for bisulfite sequencing. *Gigascience* 4:29. 10.1186/s13742-015-0070-9 26140213 PMC4488126

[B44] Gibson D. J. Connolly J. Hartnett D. C. Weidenhamer D. (2004). Designs for greenhouse studies of interactions between plants. *J. Ecol.* 87 1–16. 10.1046/j.1365-2745.1999.00321.x

[B45] González-Benito M. E. Ibáñez M. Á. Pirredda M. Mira S. Martín C. (2020). Application of the MSAP Technique to Evaluate Epigenetic Changes in Plant Conservation. *Int. J. Mol. Sci*. 21:7459. 10.3390/ijms21207459 33050382 PMC7589462

[B46] Gourcilleau D. Bogeat-Triboulot M. B. Le Thiec D. Lafon-Placette C. Delaunay A. El-Soud (2010). DNA methylation and histone acetylation: genotypic variations in hybrid poplars, impact of water deficit and relationships with productivity. *Ann. For. Sci.* 67:208. 10.1051/forest/2009101

[B47] Graña O. López-Fernández H. Fdez-Riverola F. Pisano D. Glez-Peña D. (2017). bicycle: a bioinformatics pipeline to analyze bisulfite sequencing data. *Bioinform* 34 1414–1415. 10.1093/bioinformatics/btx778 29211825

[B48] Grehl C. Wagner M. Lemnian I. Glaser B. Grosse I. (2020). Performance of Mapping Approaches for Whole-Genome Bisulfite Sequencing Data in Crop Plants. *Front. Plant Sci.* 11:176. 10.3389/fpls.2020.00176 32256504 PMC7093021

[B49] Gugger P. F. Fitz-Gibbon S. PellEgrini M. Sork V. L. (2016). Species-wide patterns of DNA methylation variation in *Quercus lobata* and their association with climate gradients. *Mol. Ecol.* 25 1665–1680. 10.1111/mec.13563 26833902

[B50] Hasbún R. Iturra C. Bravo S. Rebolledo-Jaramillo B. Valledor L. (2016). Differential Methylation of Genomic Regions Associated with Heteroblasty Detected by M&M Agorithm in the Nonmodel Species *Eucalyptus globulus* Labill. *Int. J. Genom.* 2016:4395153. 10.1155/2016/4395153 27123440 PMC4829722

[B51] Herrera C. M. Bazaga P. (2013). Epigenetic correlates of plant phenotypic plasticity: DNA methylation differs between prickly and nonprickly leaves in heterophyllous *Ilex aquifolium* (Aquifoliaceae) trees. *Bot. J. Linn. Soc.* 171 441–452. 10.1111/boj.12007

[B52] IPCC (2018). *Global warming of 1.5^°^C. An IPCC special report on the impacts of global warming of 1.5^°^C above pre-industrial levels and related global greenhouse gas emission pathways, in the context of strengthening the global response to the threat of climate change, sustainable development, and efforts to eradicate poverty.* Geneva: World Meteorological Organization.

[B53] Iwasaki M. Paszkowski J. (2014). Epigenetic memory in plants. *EMBO J.* 33 1–12. 10.15252/embj.201488883 25104823 PMC4195768

[B54] Jump A. S. Peñuelas J. (2005). Running to stand still: adaptation and the response of plants to rapid climate change. *Ecol. Lett.* 8 1010–1020. 10.1111/j.1461-0248.2005.00796.x 34517682

[B55] Karan R. DeLeon T. Biradar H. Subudhi P. K. (2012). Salt Stress Induced Variation in DNA Methylation Pattern and Its Influence on Gene Expression in Contrasting Rice Genotypes. *PLoS One* 7:e40203. 10.1371/journal.pone.0040203 22761959 PMC3386172

[B56] Kijowska-Oberc J. Staszak A. M. Kamiński J. Ratajczak E. (2020). Adaptation of Forest Trees to Rapidly Changing Climate. *Forests* 11:123. 10.3390/f11020123

[B57] Kinlaw C. S. Neale D. B. (1997). Complex gene families in pine genomes. *Trends Plant Sci.* 2 356–359. 10.1016/S1360-1385(97)84624-9

[B58] Kinoshita T. Seki M. (2014). Epigenetic memory for stress response and adaptation in plants. *Plant Cell Physiol*. 55 1859–1863. 10.1093/pcp/pcu125 25298421

[B59] Korotko U. Chwialkowska K. Sanki-Sawczenko I. Kwasmiewski M. (2021). DNA Demethylation in Response to Heat Stress in *Arabidopsis thaliana*. *Int. J. Mol. Sci.* 22:1555. 10.3390/ijms22041555 33557095 PMC7913789

[B60] Krueger F. Andrews S. R. (2011). Bismark: a flexible aligner and methylation caller for Bisulfite-seq applications. *Bioinform* 27 1571–1572. 10.1093/bioinformatics/btr167 21493656 PMC3102221

[B61] Kurdyukov S. Bullock M. (2016). DNA methylation analysis: choosing the right method. *Biology* 5:3. 10.3390/biology5010003 26751487 PMC4810160

[B62] Kuzmin D. A. Feranchuk S. I. Sharov V. V. Cybin A. N. Makolov S. V. Putintseva Y. A. (2019). Stepwise large genome assembly approach: a case of Siberian larch (*Larix sibirica* Ledeb). *BMC Bioinf.* 20:37. 10.1186/s12859-018-2570-y 30717661 PMC6362582

[B63] Kvaalen H. Johnsen Ø (2008). Timing of bud set in *Picea abies* is regulated by a memory of temperature during zygotic and somatic embryogenesis. *New Phytol.* 177 49–59. 10.1111/j.1469-8137.2007.02222.x 17924949

[B64] Lafon-Placette C. Le Gac A. L. Chauveau D. Segura V. Delaunay A. Lesage-Descauses M. C. (2018). Changes in the epigenome and transcriptome of the poplar shoot apical meristem in response to water availability affect preferentially hormone pathways. *J. Exp. Bot.* 69 537–551. 10.1093/jxb/erx409 29211860

[B65] Lamelas L. Valledor L. Escandón M. Pinto G. Cañal M. J. Meijon M. (2020). Integrative analysis of the nuclear proteome in *Pinus radiata* reveals thermopriming coupled to epigenetic regulation. *J. Exp. Bot.* 71 2040–2057. 10.1093/jxb/erz524 31781741 PMC7094079

[B66] Lang-Mladek C. Popova O. Kiok K. Berlinger M. Rakic B. Aufsatz W. (2010). Transgenerational inheritance and resetting of stress-induced loss of epigenetic gene silencing in *Arabidopsis*. *Mol. Plant* 3 594–602. 10.1093/mp/ssq014 20410255 PMC2877484

[B67] Law J. A. Jacobsen S. E. (2010). Establishing, maintaining and modifying DNA methylation patterns in plants and animals. *Nat. Rev. Genet.* 11 204–220. 10.1038/nrg2719 20142834 PMC3034103

[B68] Le Gac A. L. Lafon-Placette C. Chauveau D. Segura V. Delaunay A. Fichot R. (2018). Winter-dormant shoot apical meristem in poplar trees shows environmental epigenetic memory. *J. Exp. Bot.* 69 4821–4837. 10.1093/jxb/ery271 30107545 PMC6137975

[B69] Li D. Zhang B. Xing X. Wang T. (2015). Combining MeDIP-seq and MRE-seq to investigate genome-wide CpG methylation. *Methods* 72 29–40. 10.1016/j.ymeth.2014.10.032 25448294 PMC4300244

[B70] Li R. Hu F. Li B. Zhang Y. Chen M. Fan T. (2020). Whole genome bisulfite sequencing methylome analysis of mulberry (*Morus alba*) reveals epigenome modifications in response to drought stress. *Sci. Rep.* 10:8013. 10.1038/s41598-020-64975-5 32415195 PMC7228953

[B71] Li W. Lin Y. C. Li Q. Shi R. Lin C.-Y. Chen H. (2014). A robust chromatin immunoprecipitation protocol for studying transcription factor–DNA interactions and histone modifications in wood-forming tissue. *Nat. Protoc.* 9 2180–2193. 10.1038/nprot.2014.146 25144269

[B72] Li Y. Dong X. M. Jin F. Shen Z. Chao Q. Wang B. C. (2017). Histone Acetylation Modifications Affect Tissue-Dependent Expression of Poplar Homologs of C4 Photosynthetic Enzyme Genes. *Front. Plant Sci.* 8:950. 10.3389/fpls.2017.00950 28642769 PMC5462996

[B73] Liang D. Zhang Z. Wu H. Huang C. Shuai P. Ye C. Y. (2014). Single-base-resolution methylomes of *Populus trichocarpa* reveal the association between DNA methylation and drought stress. *BMC Genet.* 15:S9. 10.1186/1471-2156-15-S1-S9 25080211 PMC4118614

[B74] Lin X. Sun D. Rodriguez B. Zhao Q. Sun H. Zhang Y. (2013). BSeQC: quality control of bisulfite sequencing experiments. *Bioinform.* 29 3227–3229. 10.1093/bioinformatics/btt548 24064417 PMC3842756

[B75] Linares J. C. Camarero J. J. Carreira J. A. (2010). Competition modulates the adaptation capacity of forests to climatic stress: insights from recent growth decline and death in relict stands of the Mediterranean fir *Abies pinsapo*. *J. Ecol.* 98 592–603. 10.1111/j.1365-2745.2010.01645.x

[B76] Linares J. C. Taïqui L. Camarero J. J. (2011). Increasing Drought Sensitivity and Decline of Atlas Cedar (*Cedrus atlantica*) in the Moroccan Middle Atlas Forests. *Forests* 2 777–796. 10.3390/f2030777

[B77] Ma X. Zhang C. Zhang B. Yang C. Li S. (2016). Identification of genes regulated by histone acetylation during root development in *Populus trichocarpa*. *BMC Genom.* 17:96. 10.1186/s12864-016-2407-x 26847576 PMC4743431

[B78] Macías M. Andreu L. Bosch O. Camarero J. J. Gutiérrez E. (2006). Increasing aridity is enhancing silver fir *Abies alba* (Mill.) water stress in its south-western distribution limit. *Clim. Change* 79, 289–313. 10.1007/s10584-006-9071-0

[B79] Mackay J. Dean J. F. Plomion C. Peterson D. G. Cánovas F. M. Pavy N. (2012). Towards decoding the conifer giga-genome. *Plant Mol. Biol.* 80 555–569. 10.1007/s11103-012-9961-7 22960864

[B80] Markus C. Pecinka A. Merotto A. Jr. (2021). Insights into the Role of Transcriptional Gene Silencing in Response to Herbicide-Treatments in *Arabidopsis thaliana*. *Int. J. Mol. Sci.* 22:3314. 10.3390/ijms22073314 33804990 PMC8037345

[B81] Martin A. Troadec C. Boualem A. Rajab M. Fernandez R. Morin H. (2009). A transposon-induced epigenetic change leads to sex determination in melon. *Nature* 461 1135–1138. 10.1038/nature08498 19847267

[B82] Martin M. (2011). Cutadapt removes adapter sequences from high-throughput sequencing reads. *EMBnet J.* 17 10–12. 10.14806/ej.17.1.200

[B83] Mascher M. Wicker T. Jenkins J. Plott C. Lux T. Koh C. S. (2021). Long-read sequence assembly: a technical evaluation in barley. *Plant Cell* 33 1888–1906. 10.1093/plcell/koab077 33710295 PMC8290290

[B84] Maunakea A. K. Nagarajan R. P. Bilenky M. Ballinger T. J. D’Souza C. Fouse S. D. (2010). Conserved role of intragenic DNA methylation in regulating alternative promoters. *Nature* 466 253–257. 10.1038/nature09165 20613842 PMC3998662

[B85] McDowell N. G. Williams A. P. Xu C. Pockman W. T. Dickman L. T. Sevanto S. (2016). Multi-scale predictions of massive conifer mortality due to chronic temperature rise. *Nat. Clim. Change* 6 295–300. 10.1038/nclimate2873

[B86] Merce C. Bayer P. E. Fernandez C. T. Batley J. Edwards D. (2020). Induced Methylation in Plants as a Crop Improvement Tool: Progress and Perspectives. *Agronomy* 10:1484. 10.3390/agronomy10101484

[B87] Miryeganeh M. Marlétaz F. Gavriouchkina D. Saze H. (2021). De novo genome assembly and in natura epigenomics reveal salinity-induced DNA methylation in the mangrove tree *Bruguiera gymnorhiza*. *New Phytol*. 2021:17738. 10.1111/nph.17738 34532854 PMC9293310

[B88] Mosca E. Cruz F. Gómez-Garrido J. Bianco L. Rellstab C. Brodbeck S. (2019). A Reference Genome Sequence for the European Silver Fir (*Abies alba* Mill.): A Community-Generated Genomic Resource. *G3* 9 2039–2049. 10.1534/g3.119.400083 31217262 PMC6643874

[B89] Mougninot P. Aparicio N. L. Gourcilleau D. Latutrie M. Marin S. Hemptinne J. L. (2021). Phenotypic Response to Light Versus Shade Associated with DNA Methylation Changes in Snapdragon Plants (*Antirrhinum majus*). *Genes* 12:227. 10.3390/genes12020227 33557416 PMC7914928

[B90] Muhammad I. I. Kong S. L. Akmar Abdullah S. N. Munusamy U. (2020). RNA-seq and ChIP-seq as Complementary Approaches for Comprehension of Plant Transcriptional Regulatory Mechanism. *Int. J. Mol. Sci.* 21:167. 10.3390/ijms21010167 31881735 PMC6981605

[B91] Neale D. B. McGuire P. E. Wheeler N. C. Stevens K. A. Crepeau M. W. Cardeno C. (2017). The Douglas-Fir Genome Sequence Reveals Specialization of the Photosynthetic Apparatus in Pinaceae. *G3* 7 3157–3167. 10.1534/g3.117.300078 28751502 PMC5592940

[B92] Neale D. B. Wegrzyn J. L. Stevens K. A. Zimin A. V. Puiu D. Crepeau M. W. (2014). Decoding the massive genome of loblolly pine using haploid DNA and novel assembly strategies. *Genome Biol.* 15:R59. 10.1186/gb-2014-15-3-r59 24647006 PMC4053751

[B93] Nystedt B. Street N. R. Wetterbom A. Zuccolo A. Lin Y.-C. Scofield D. G. (2013). The Norway spruce genome sequence and conifer genome evolution. *Nature* 497 579–584. 10.1038/nature12211 23698360

[B94] Park Y. Wu H. (2016). Differential methylation analysis for BS-seq data under general experimental design. *Bioinform* 32 1446–1453. 10.1093/bioinformatics/btw026 26819470 PMC12157722

[B95] Paun O. Verhoeven K. J. F. Richards L. (2019). Opportunities and limitations of reduced representation bisulfite sequencing in plant ecological epigenomics. *New Phytol.* 221 738–742. 10.1111/nph.15388 30121954 PMC6504643

[B96] Pereira W. J. Pappas M. D. C. R. Grattapaglia D. Pappas G. J. Jr. (2020). A cost-effective approach to DNA methylation detection by Methyl Sensitive DArT sequencing. *PLoS One* 15:e0233800. 10.1371/journal.pone.00233800 32497070 PMC7272069

[B97] Perrin A. Daccord N. Roquis D. Celton J. M. Vergne E. Bucher E. (2020). Divergent DNA Methylation Signatures of Juvenile Seedlings, Grafts and Adult Apple Trees. *Epigenomes* 4:4. 10.3390/epigenomes4010004 34968238 PMC8594697

[B98] Petit R. J. Hampe A. (2006). Some Evolutionary Consequences of Being a Tree. *Annu. Rev. Ecol. Evol. Syst.* 37 187–214. 10.1146/annurev.ecolsys.37.091305.110215

[B99] Raj S. Bräutigam K. Hamanishi E. T. Wilkins O. Thomas B. R. Schroeder W. (2011). Clone history shapes *Populus* drought responses. *PNAS* 108 12521–12526. 10.1073/pnas.1103341108 21746919 PMC3145742

[B100] Rapp R. A. Wendel J. F. (2005). Epigenetics and plant evolution. *New Phytol.* 168 81–91. 10.1111/j.1469-8137.2005.01491.x 16159323

[B101] Rauluseviciute I. Drabløs F. Rye M. B. (2019). DNA methylation data by sequencing: experimental approaches and recommendations for tools and pipelines for data analysis. *Clin. Epigenet.* 11:193. 10.1186/s13148-019-0795-x 31831061 PMC6909609

[B102] Reyna-López G. A. Simpson J. Ruiz-Herrera J. (1997). Differences in DNA methylation patterns are detectable during the dimorphic transition of fungi by amplification of restriction polymorphisms. *Mol. Gen. Genet.* 253 703–710. 10.1007/s004380050374 9079881

[B103] Rico L. Ogaya R. Barbeta A. Peñuelas J. (2014). Changes in DNA methylation fingerprint of *Quercus ilex* trees in response to experimental field drought simulating projected climate change. *Plant Biol.* 16 419–427. 10.1111/plb.12049 23889779

[B104] Robinson M. D. McCarthy D. J. Smyth G. K. (2010). edgeR: a Bioconductor package for differential expression analysis of digital gene expression data. *Bioinform.* 26 139–140. 10.1093/bioinformatics/btp616 19910308 PMC2796818

[B105] Rothkegel K. Espinoza A. Sanhueza D. Lillo-Carmona V. Riveros A. Campos-Vargas R. (2021). Identification of DNA Methylation and Transcriptomic Profiles Associated With Fruit Mealiness in *Prunus persica* (L.) Batsch. *Front. Plant sci.* 12:684130. 10.3389/fpls.2021.684130 34178003 PMC8222998

[B106] Sáez-Laguna E. Guevara M. A. Díaz L. M. Sánchez-Gómez D. Collada C. Sánchez-Gómez D. (2014). Epigenetic Variability in the Genetically Uniform Forest Tree Species *Pinus pinea* L. *PLoS One* 9:e103145. 10.1371/journal.pone.0103145 25084460 PMC4118849

[B107] Sánchez-Salguero R. Ortíz C. Covelo F. Ochoa V. García-Ruíz R. Seco J. I. (2015). Regulation of Water Use in the Southernmost European Fir (*Abies pinsapo* Boiss.): Drought Avoidance Matters. *Forests* 6 2241–2260. 10.3390/f6062241

[B108] Schield D. R. Wlash M. R. Card D. C. Andrew A. l. Adams R. H. Castoe T. A. (2016). EpiRADseq: scalable analysis of genomewide patterns of methylation using next-generation sequencing. *Methods Ecol. Evol.* 7 60–69. 10.1111/2041-210X.12435

[B109] Schönberger B. Chen X. Mager S. Ludewig U. (2016). Site-Dependent Differences in DNA Methylation and Their Impact on Plant Establishment and Phosphorus Nutrition in *Populus trichocarpa*. *PLoS One* 11:e0168623. 10.1371/journal.pone.0168623 27992519 PMC5167412

[B110] Song Y. Ci D. Tian M. Zhang D. (2016). Stable methylation of a non-coding RNA gene regulates gene expression in response to abiotic stress in *Populus simonii*. *J. Exp. Bot.* 67 1477–1492. 10.1093/jxb/erv543 26712827

[B111] Sow M. D. Allona I. Ambroise C. Conde D. Fichot R. Gribkova S. (2018). “Epigenetics in Forest Trees: State of the Art and Potential Implications for Breeding and Management in a Context of Climate Change,” in *Advances in Botanical Research*, eds Mirouze M. Bucher E. Gallusci P. (Cambridge: Academic Press), 387–453.

[B112] Sow M. D. Le Gac A. L. Fichot R. Lanciano S. Delaunay A. Le Jan I. (2021). RNAi suppression of DNA methylation affects the drought stress response and genome integrity in transgenic poplar. *New Phytol*. 2021:17555. 10.1111/nph.17555 34128549

[B113] Stevens K. A. Wegrzyn J. L. Zimin A. Puiu D. Crepeau M. Cardeno C. (2016). Sequence of the Sugar Pine Megagenome. *Genetics* 204 1613–1626. 10.1534/genetics.116.193227 27794028 PMC5161289

[B114] Su Y. Bai X. Yang W. Wang W. Chen Z. Ma J. (2018). Single-base-resolution methylomes of *Populus euphratica* reveal the association between DNA methylation and salt stress. *Tree Genet. Genomes* 14:86. 10.1007/s11295-018-1298-1

[B115] Szcześniak M. W. Rosikiewicz W. Makalowska I. (2015). CANTATAdb: A collection of plant long non-coding RNAs. *Plant Cell Physiol*. 57:e8. 10.1093/pcp/pcv201 26657895 PMC4722178

[B116] Takuno S. Ran J. H. Gaut B. (2016). Evolutionary patterns of genic DNA methylation vary across land plants. *Nat. Plants* 2:15222. 10.1038/nplants.2015.222 27249194

[B117] Thu K. L. Vucic E. A. Kennett J. Y. Heryet C. Brown C. J. Lam W. L. (2009). Methylated DNA immunoprecipitation. *J. Vis. Exp.* 23:935. 10.3791/935 19241501 PMC2763296

[B118] Trucchi E. Mazzarella A. B. Gilfillan G. D. Lorenzo M. T. Schönswetter P. Paun O. (2016). BsRADseq: screening DNA methylation in natural populations of non-model species. *Mol. Ecol.* 25 1697–1713. 10.1111/mec.13550 26818626 PMC4949719

[B119] Trugman A. Anderegg L. D. L. Anderegg W. R. L. Das A. J. Stephenson N. L. (2021). Why is tree drought mortality so hard to predict? *Trends Ecol. Evol.* 36 520–532. 10.1016/j.tree.2021.02.001 33674131

[B120] van Gurp T. Wagemaker N. C. A. M. Wouters B. Vergeer P. Ouborg J. N. J. Verhoeven K. J. F. (2016). epiGBS: reference-free reduced representation bisulfite sequencing. *Nat. Methods* 13 322–324. 10.1038/nmeth.3763 26855363

[B121] Vanden Broeck A. Cox K. Brys R. Castiglione S. Cicatelli A. Guarino F. (2018). Variability in DNA methylation and generational plasticity in the lombardy poplar, a single genotype worldwide distributed since the eighteenth century. *Front. Plant Sci.* 9:1635. 10.3389/fpls.2018.01635 30483290 PMC6242946

[B122] Wang W. S. Pan Y. J. Zhao X. Q. Dwivedi D. Zhu L. H. Ali J. (2010). Drought-induced site-specific DNA methylation and its association with drought tolerance in rice (*Oryza sativa* L.). *J. Exp. Bot.* 62 1951–1960. 10.1093/jxb/erq391 21193578 PMC3060682

[B123] Wang Z. Gerstein M. Snyder M. (2009). RNA-Seq: a revolutionary tool for transcriptomics. *Nat. Rev. Genet.* 10 57–63. 10.1038/nrg2484 19015660 PMC2949280

[B124] Wang Z. Xianfeng L. Jiang Y. Shao Q. Liu Q. Chen B. Y. (2015). swDMR: A Sliding Window Approach to Identify Differentially Methylated Regions Based on Whole Genome Bisulfite Sequencing. *PLoS One* 10:e0132866. 10.1371/journal.pone.0132866 26176536 PMC4503785

[B125] Warren R. L. Keeling C. I. Yuen M. M. Raymond A. Taylor G. A. Vandervalk B. P. (2015). Improved white spruce (*Picea glauca*) genome assemblies and annotation of large gene families of conifer terpenoid and phenolic defense metabolism. *Plant J.* 83 189–212. 10.1111/tpj.12886 26017574

[B126] Webber J. Ott P. Owens J. Binder W. (2005). Elevated temperature during reproductive development affects cone traits and progeny performance in *Picea glauca* x *engelmannii* complex. *Tree Physiol.* 25 1219–1227. 10.1093/treephys/25.10.1219 16076771

[B127] Wei J.-W. Huang K. Yang C. Kang C.-S. (2016). Non-coding RNAs as regulators in epigenetics. *Oncol. Rep.* 37 3–9. 10.3892/or.2016.5236 27841002

[B128] Werner O. Prudencio A. S. de la Cruz-Martínez E. I. Nieto-Liglde M. Martínez-Gómez P. Ros R. M. (2020). A cost reduced variant of epi-genotyping by sequencing for studying DNA methylation in non-model organisms. *Front. Plant Sci.* 11:694. 10.3389/fpls.2020.00694 32547585 PMC7270828

[B129] West-Eberhard M. J. (2008). “Phenotypic Plasticity,” in *Encyclopedia of Ecology*, eds Erik Jørgensen S. Fath B. D. (Cambridge: Academic Press), 2701–2707. 10.1016/B978-008045405-4.00837-5

[B130] Wilson G. A. Dhami P. Feber A. Cortazar D. Suzuki Y. Schulz R. (2012). Resources for methylome analysis suitable for gene knockout studies of potential epigenome modifiers. *GigaScience* 1:3. 10.1186/2047-217X-1-3 23587164 PMC3617451

[B131] Wreczycka K. Gosdschan A. Yusuf D. Grüning B. Assenov Y. Akalin A. (2017). Strategies for analyzing bisulfite sequencing data. *J. Biotechnol.* 216 105–115. 10.1016/j.jbiotec.2017.08.007 28822795

[B132] Xi Y. Li W. (2009). BSMAP: whole genome bisulfite sequence MAPping program. *BMC Bioinform*. 10:232. 10.1186/1471-2105-10-232 19635165 PMC2724425

[B133] Xiong Z. Xu C. G. Saghai Maroof M. A. Zhang Q. (1999). Patterns of cytosine methylation in an elite rice hybrid and its parental lines, detected by a methylation-sensitive amplification polymorphism technique. *Mol. Gen. Genet*. 261 439–446. 10.1007/s004380050986 10323223

[B134] Xu J. Zhou S. Gong X. Song Y. van Nocker S. Ma F. (2018). Single-base methylome analysis reveals dynamic epigenomic differences associated with water deficit in apple. *Plant Biotechnol. J.* 16 672–687. 10.1111/pbi.12820 28796917 PMC5787839

[B135] Yakovlev I. Fossdal C. G. Skrøppa T. Olsen J. E. Jahren A. H. Johnsen O. (2012). An adaptive epigenetic memory in conifers with important implications for seed production. *Seed Sci. Res.* 22 63–76. 10.1017/s0960258511000535

[B136] Yakovlev I. A. Carneros E. Lee Y. Olsen J. E. Fossdal C. G. (2016). Transcriptional profiling of epigenetic regulators in somatic embryos during temperature induced formation of an epigenetic memory in Norway spruce. *Planta* 243 1237–1249. 10.1007/s00425-016-2484-8 26895338

[B137] Yakovlev I. A. Lee Y. Rotter B. Olsen J. E. Skrøppa T. Johnsen O. (2014). Temperature-dependent differential transcriptomes during formation of an epigenetic memory in Norway spruce embryogenesis. *Tree Genet. Genomes* 10 355–366. 10.1007/s11295-013-0691-z

[B138] Zemach A. McDaniel I. E. Silva P. Ziberman D. (2010a). Genome-wide evolutionary analysis of eukaryotic DNA methylation. *Science* 328 916–919. 10.1126/science.1186366 20395474

[B139] Zemach A. Kim M. Y. Silva P. Rodrigues J. A. Dotson B. Brooks M. D. (2010b). Local DNA hypomethylation activates genes in rice endosperm. *PNAS* 107 18729–18734. 10.1073/pnas.1009695107 20937895 PMC2972920

[B140] Zemach A. Ziberman D. (2010). Evolution of eukaryotic DNA methylation and the pursuit of saver sex. *Curr. Biol.* 20 R780–R785. 20833323 10.1016/j.cub.2010.07.007

[B141] Zhang H. Lang Z. Zhu J. K. (2018). Dynamics and function of DNA methylation in plants. *Nat. Rev. Mol.* 19 489–506. 10.1038/s41580-018-0016-z 29784956

[B142] Zhao J. G. Lu Z. G. Wang L. Jin B. (2021). Plant responses to heat stress: physiology, transcription, noncoding RNAs and epigenetics. *Int. J. Mol. Sci.* 22:117. 10.3390/ijms22010117 33374376 PMC7795586

[B143] Zhao M. T. Whyte J. J. Hopkins G. M. Kirk M. D. Prather R. S. (2014). Methylated DNA immunoprecipitation and high-throughput sequencing (MeDIP-seq) using low amounts of genomic DNA. *Cell Reprogram* 16 175–184. 10.1089/cell.2014.0002 24773292

[B144] Zheng X. Chen L. Li M. Lou Q. Xia H. Wang P. (2013). Transgenerational Variations in DNA Methylation Induced by Drought Stress in Two Rice Varieties with Distinguished Difference to Drought Resistance. *PLoS One* 8:e80253. 10.1371/journal.pone.0080253 24244664 PMC3823650

